# Platinum Complexes in Colorectal Cancer and Other Solid Tumors

**DOI:** 10.3390/cancers13092073

**Published:** 2021-04-25

**Authors:** Beate Köberle, Sarah Schoch

**Affiliations:** 1 Department of Food Chemistry and Toxicology, Karlsruhe Institute of Technology, Adenauerring 20a, 76131 Karlsruhe, Germany; 2 Department of Laboratory Medicine, Lund University, Scheelevägen 2, 223 81 Lund, Sweden; sarah.schoch@med.lu.se

**Keywords:** platinum drugs, colorectal cancer, mismatch repair defect, p53 signaling

## Abstract

**Simple Summary:**

Cisplatin is successfully used for the treatment of various solid cancers. Unfortunately, it shows no activity in colorectal cancer. The resistance phenotype of colorectal cancer cells is mainly caused by alterations in p53-controlled DNA damage signaling and/or defects in the cellular mismatch repair pathway. Improvement of platinum-based chemotherapy in cisplatin-unresponsive cancers, such as colorectal cancer, might be achieved by newly designed cisplatin analogues, which retain activity in unresponsive tumor cells. Moreover, a combination of cisplatin with biochemical modulators of DNA damage signaling might sensitize cisplatin-resistant tumor cells to the drug, thus providing another strategy to improve cancer therapy.

**Abstract:**

Cisplatin is one of the most commonly used drugs for the treatment of various solid neoplasms, including testicular, lung, ovarian, head and neck, and bladder cancers. Unfortunately, the therapeutic efficacy of cisplatin against colorectal cancer is poor. Various mechanisms appear to contribute to cisplatin resistance in cancer cells, including reduced drug accumulation, enhanced drug detoxification, modulation of DNA repair mechanisms, and finally alterations in cisplatin DNA damage signaling preventing apoptosis in cancer cells. Regarding colorectal cancer, defects in mismatch repair and altered p53-mediated DNA damage signaling are the main factors controlling the resistance phenotype. In particular, p53 inactivation appears to be associated with chemoresistance and poor prognosis. To overcome resistance in cancers, several strategies can be envisaged. Improved cisplatin analogues, which retain activity in resistant cancer, might be applied. Targeting p53-mediated DNA damage signaling provides another therapeutic strategy to circumvent cisplatin resistance. This review provides an overview on the DNA repair pathways involved in the processing of cisplatin damage and will describe signal transduction from cisplatin DNA lesions, with special attention given to colorectal cancer cells. Furthermore, examples for improved platinum compounds and biochemical modulators of cisplatin DNA damage signaling will be presented in the context of colon cancer therapy.

## 1. Introduction

**Figure 1:**
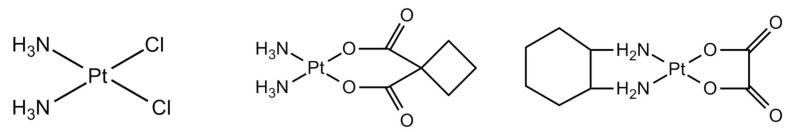
Chemical structures of the clinically approved platinum-based anticancer drugs cisplatin, carboplatin, and oxaliplatin.

Cisplatin (*cis*-diamminedichloro-platinum (II)) (Figure 1) is one of the most active chemotherapeutic drugs available to treat a variety of malignancies. It is particularly effective in the treatment of testicular germ cell tumors with response rates above 90%, but also displays clinical activity against bladder, ovarian, head and neck, cervical, and lung cancer [1,2,3]. The mechanisms by which the antitumor activity of cisplatin is achieved have been studied extensively in various cancer cells, and there is strong evidence that the cytotoxicity of the drug is mediated by its interaction with DNA resulting in DNA lesions which may inhibit both DNA transcription and replication [4,5]. Cisplatin enters the cells by both passive diffusion through the plasma membrane and by active transport mediated by several membrane transporters [6,7]. Once inside the cell, the two labile chloride leaving groups of cisplatin are replaced by water resulting in activation of the drug [8]. The aquated form of cisplatin forms covalent bonds with the N7 position of guanine and adenine resulting in DNA crosslinks between two purine bases. Most crosslinks are formed on the same strand of DNA, such as GpG 1,2-intrastrand crosslinks, ApG 1,2-intrastrand crosslinks and, to a lesser extent, GpXpG 1,3-intrastrand crosslinks. Crosslinks between two guanine bases on the opposite strands of DNA (DNA ICLs) are less abundant accounting for 1-2% of cisplatin-induced lesions [9,10]. In response to platinum DNA damage, cells activate various signal transduction pathways which either induce cell cycle arrest and therefore facilitate DNA repair or trigger apoptosis and hence cell death [11,12]. Central in the DNA damage response is the tumor suppressor protein p53, which is phosphorylated and activated upon treatment with cisplatin [13].

Despite the successful treatment of various tumor entities, colorectal cancer has been shown to be unresponsive to cisplatin, and this resistance has limited the therapeutic success. Cisplatin resistance in tumor cells is frequently observed and might be either intrinsic to the tumor, as it is the case for pancreatic and colorectal carcinoma or might develop during courses of therapy, as observed, for example, for ovarian cancer [11]. Potential mechanisms underlying tumor cell resistance have been identified in various in vitro models of cisplatin resistant tumor cell lines and include changes in drug transport leading to reduced drug accumulation, enhanced drug detoxification by thiol-containing molecules, such as glutathione and metallothionein, altered DNA repair mechanisms, namely nucleotide excision repair (NER), ICL repair and DNA mismatch repair (MMR), and finally alterations in the signal transduction pathways, which in turn will allow cancer cells to evade cisplatin-induced cell death [14,15]. The resistance mechanisms appear to be cell line dependent, hence a particular tumor may exhibit one or more of the described mechanisms of resistance.

The tumor suppressor protein p53 has a central role in cisplatin damage signaling and controls down-stream events, such as cell cycle arrest/DNA repair or apoptosis [12]. Therefore, inactivation or mutation of p53 alters the cytotoxicity of cisplatin [16,17]. In colorectal cancer, disruption of p53 damage signaling as well as MMR deficiency appear to be main determinants underlying the observed drug resistance [18,19]. The present review describes cisplatin resistance in colorectal cancer, with a special emphasis given to the p53 status and MMR repair as main contributors to the resistance phenotype. Approaches to overcome the disadvantage of cisplatin in the treatment of unresponsive cancers include administration of cisplatin-derived compounds with activity in resistant cancer and application of drugs which interfere with p53-mediated signaling and will be described, with a special emphasis given to colorectal cancer.

## 2. Processing of Cisplatin-Induced DNA Damage in Cancer Cells

### 2.1. Removal of Cisplatin-Induced DNA Crosslinks

**Figure 2:**
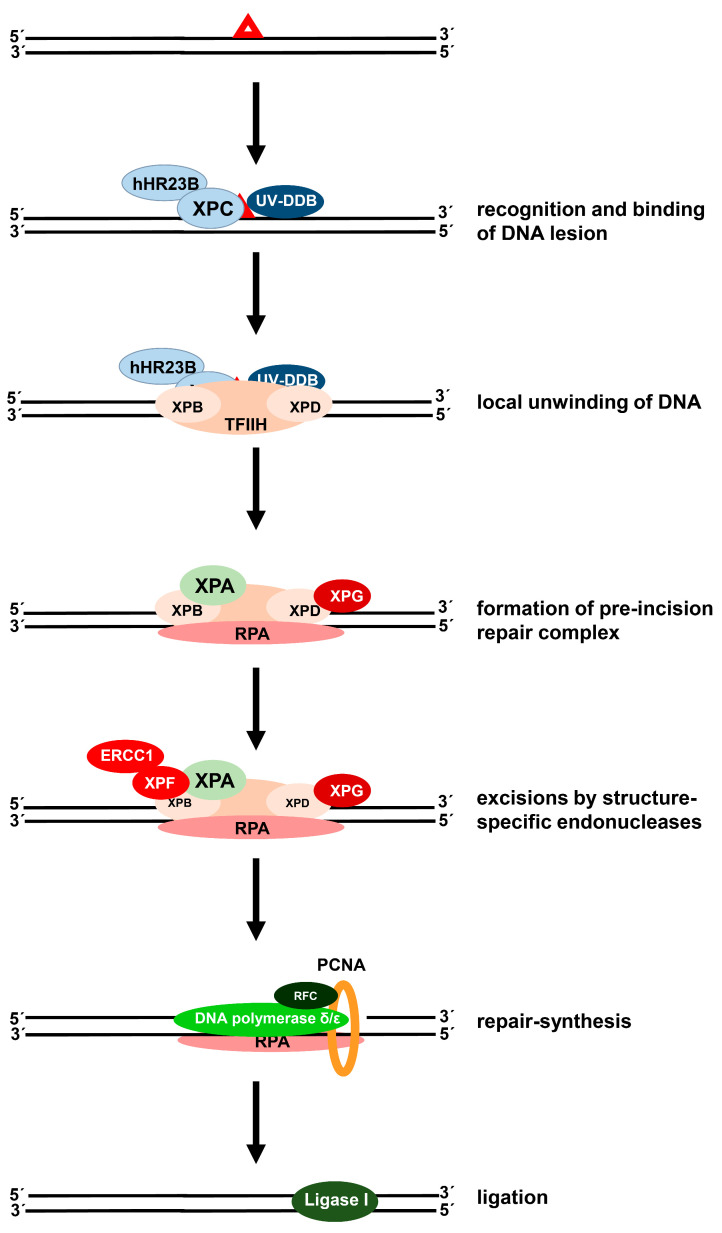
Mechanism of human nucleotide excision repair. The repair proteins XPC-hHR23B and UV-DDB detect and bind to the DNA lesion (red triangle). The transcription factor TFIIH with its helicase subunits XPB and XPD is recruited to the damage, leading to local unwinding of the DNA around the lesion. A pre-incision complex is formed by recruitment of XPA, RPA, and XPG, followed by incisions on both sides of the damage, mediated by the endonucleases XPG and ERCC1-XPF complex. DNA polymerase δ/ε, supported by PCNA and RFC, catalyze DNA re-synthesis, the nick is sealed by DNA ligase I.

As the induction of DNA damage is generally considered to be the basis of the therapeutic effect of cisplatin [8], the cellular capacity to repair DNA damage might therefore influence the antitumor activity of this drug and hence may modulate the clinical activity. Cisplatin-induced lesions are repaired by different DNA repair pathways. The predominant DNA intrastrand crosslinks are removed by NER, the main DNA repair pathway dealing with lesions altering the helical structure of DNA, such as UV-induced cyclobutane pyrimidine dimers and 6-4 photoproducts, and DNA lesions induced by many chemotherapeutic drugs (Figure 2) [20,21,22].

NER requires the action of around 30 repair proteins, which function by the stepwise assembly of several repair protein complexes at the site of the damage [23]. The NER process involves recognition of the damage by the XPC protein with its partner hHR23B, together with the DNA binding protein UV-DDB [24]. This step is followed by local unwinding of the helix by the helicases XPB and XPD, which are subunits of the transcription complex TFIIH. XPA, RPA, and XPG are recruited resulting in the formation of the pre-incision complex. Incisions are then made on both sides of the damage by the endonucleases ERCC1-XPF and XPG. A 24-32mer oligonucleotide containing the damage is released, the gap is filled by the DNA repair synthesis mediated by DNA polymerases δ and ε and supported by PCNA and RFC, and finally DNA ligase I will seal the nick [25]. The efficiency, with which the different cisplatin-induced intrastrand crosslinks are repaired, varies, most likely due to the extent of the helical distortions caused by the lesions [26]. Repair of cisplatin-induced ICLs is much more complex and a challenging problem for cells. It is postulated that different DNA repair pathways may operate together, based on experimental evidence that homologous recombination, translesion synthesis, the Fanconi anemia pathway, and NER apparently all contribute to the removal of ICLs from DNA in eukaryotic cells [27,28,29].

We and others have demonstrated a correlation between removal of cisplatin-induced DNA platination and cellular response using in vitro models of cisplatin-sensitive and -resistant tumor cell lines. While increased removal of DNA platinum damage was associated with increased resistance in ovarian cancer cells [30,31], impaired removal of ICLs was observed in cisplatin-sensitive cells derived from testicular germ cell tumors [32,33]. Regarding the tumor tissue, conclusive evidence as to whether increased removal of cisplatin damage plays a key role for the resistance phenotype observed in the clinic has not yet been presented, due to the lack of methods to measure the DNA repair activity directly in the tumor tissue. One possibility to circumvent this problem is to assess levels of DNA repair factors on the mRNA or protein level in tumor tissues and correlate the expression with response to chemotherapy. This approach revealed a correlation between the expression of the NER factor ERCC1 and clinical resistance in various cancers as head and neck squamous cell carcinoma, ovarian, lung, gastric, and bladder cancers suggesting that cisplatin resistant tumors might have enhanced DNA repair in comparison with normal cells [34,35,36].

For colorectal cancer and cell lines derived from this type of tumor, there are contradictory observations regarding the relationship between the capacity to repair cisplatin platinum damage and response to the drug. One study investigating a possible relationship between DNA damage repair and drug response revealed that siRNA mediated downregulation of the damage recognition NER protein XPC increased cisplatin sensitivity in colon cancer cells, while overexpression of XPC led to an increase in resistance [37]. Contradictory to these data, Hu et al. reported that siRNA mediated downregulation of XPC was associated with increased cisplatin resistance of two colon cancer cell lines and XPC overexpression led to drug sensitivity [38]. However, whether the manipulation of XPC levels in these studies had any functional impact on the DNA repair capacity had not been investigated. For colorectal cancer cell lines, it is therefore still controversial whether there is a causal relationship between the cellular capacity to repair cisplatin lesions and response to the drug.

Regarding the colon cancer tissue, information on the removal of cisplatin DNA damage is lacking. Rather, attempts were also made to correlate the expression levels of NER proteins with response to chemotherapy in colorectal cancer patients, however with mixed results. Regarding ERCC1, low expression levels were correlated with better survival [39], while for XPA an association between high expression levels with longer overall survival was observed [40]. Furthermore, a number of studies have attempted to identify an association between genetic alterations in repair genes and cancer treatment, with a special emphasis given to NER genes. The most frequent type of genetic alterations between individuals are single nucleotide polymorphisms (SNPs), which may lead to an amino acid substitution and in turn might alter the protein function. Such a change in the protein sequence could be functionally relevant and therefore could be associated with therapy failure or success. However, no clear association between NER SNPs and cancer treatment response in patients with colorectal cancer has been established to date [41]. It is further postulated that SNPs not only affect the response to cancer treatment, but also increase the susceptibility to carcinogenesis. A number of studies investigated whether specific NER gene SNPs are associated with an incidence for colorectal cancer, but the data are inconsistent and do not allow any conclusion as to whether a given NER gene SNP might serve as a marker for genetic susceptibility to colon cancer carcinogenesis [42,43].

### 2.2. DNA Mismatch Repair: Status in Colorectal Cancer and Effect on Cisplatin-Induced DNA Damage

**Figure 3:**
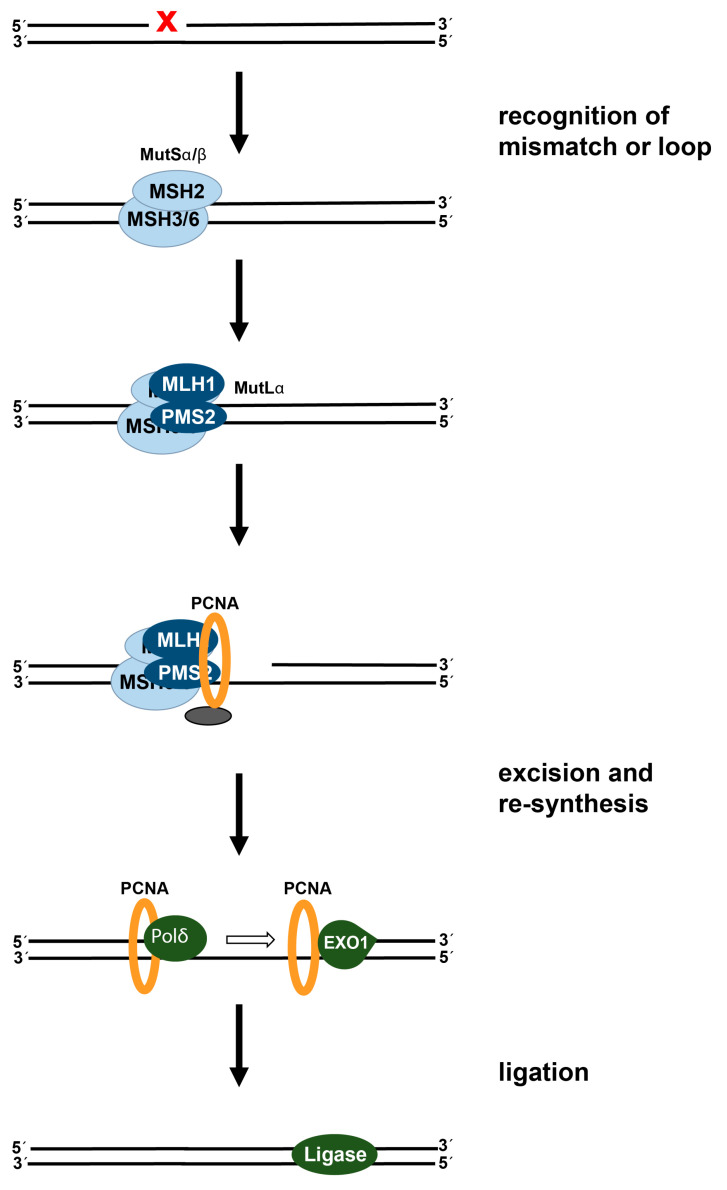
Mechanism of human mismatch repair. In addition, hMutSα/hMutSβ detect and bind to DNA mismatches or small loops. The hMutLα is recruited, followed by RFC-mediated loading of PCNA onto DNA. EXO1 binds and catalyzes excision of nucleotides beyond the mismatch/loop, followed by Polδ-catalyzed re-synthesis and DNA ligation.

In addition to NER and ICL repair, MMR is involved in the processing of cisplatin-induced DNA lesions. However, in contrast to the protective effects of NER and ICL repair, MMR potentiates the toxicity of cisplatin. MMR is a repair process, which promotes genomic stability by removing base-base mismatches, which arise during replication or as a result of DNA damage. Furthermore, looped intermediates, which are formed due to small insertions or deletions during replication, are also corrected by this repair pathway. MMR is a multistep process involving recognition of the mismatch or the looped intermediate, excision of the damage, and gap filling DNA synthesis [44,45] (Figure 3). In eukaryotes, mismatches are recognized by one of two mismatch recognition complexes, namely hMutSα and hMutSβ. In addition, hMutSα, a hererodimer consisting of hMSH2 and hMSH6, preferentially binds single base mispairs and loops of 1-2 bases, while the heterodimer hMutSβ, consisting of hMSH2 and hMSH3, more effectively binds to loops larger than two bases [46]. Upon recognition and binding of hMutSα or hMutSβ at the damaged site, the heterodimer hMutLα, consisting of MLH1 and PMS2, is recruited, resulting in a tetrameric complex [47]. Mediated by replication factor C (RFC), the proliferating cell nuclear antigen (PCNA) is loaded onto the DNA and interacts with hMutLα, which leads to activation of the endonuclease activity of PMS2 [48]. Following incision by PMS2, exonuclease 1 (EXO1) is recruited and excises the newly synthesized mismatch-containing strand, followed by gap filling by polymerase δ, and finally ligation by a DNA ligase [49]. The exact mechanism of how the MMR machinery discriminates between the parental strand and the newly synthesized strand in eukaryotic cells remains to be elucidated [44].

Mutations in MMR genes play an important role in carcinogenesis and increase cancer susceptibility [50,51]. The loss of MMR function is observed in both sporadic and hereditary colorectal cancer and is linked to mutations in either *MLH1*, *MSH2*, *MSH6* or *PMS2* [52]. Furthermore, in addition to preserving genomic integrity, intact MMR is also implicated in cytotoxicity induced by cisplatin or alkylating agents [53,54]. Various studies revealed that MMR deficient cells are considerably more tolerant to cisplatin or alkylating agents indicating that intact MMR is necessary for maximal activity of these drugs and absence of MMR is associated with increased resistance [55,56,57,58,59]. This observation might be explained by the findings that certain types of DNA lesions are recognized as mismatches by MMR proteins [60]. Regarding cisplatin-induced lesions, it was demonstrated that the MMR complex MutSα binds to cisplatin-induced intrastrand crosslinks, while ICLs are linked to processing by MutSβ [61,62,63]. Binding of the DNA lesions is followed by the execution of various pathways that can lead to apoptosis [64].

Various models have been proposed to explain how the MMR activity contributes to cisplatin toxicity. One model is based on the observations of binding of the MutSα/MutSβ complexes to cisplatin damage, which in turn will start the MMR process by recruiting MutLα. It is assumed that the MMR proteins would try to insert the “correct“ nucleotide opposite the cisplatin lesion resulting in several unsuccessful cycles of repair. Lethal intermediates will arise due to this attempted futile repair and finally trigger the apoptotic response [65]. Therefore, the loss of MMR results in reduced apoptosis and in turn resistance towards cisplatin. Another model for explaining the correlation of MMR proficiency with cisplatin sensitivity is based on observations that translesion synthesis (TLS) polymerases can bypass the 1,2-intrastrand crosslinks [66]. TLS polymerases are error prone polymerases causing mis-incorporations of bases opposite the 1,2-intrastrand crosslinks, which are targets and recognized by the MUTSα complex. Sensing of mis-incorporations in turn will cause futile repair cycles leading to DNA damage response and finally apoptosis.

An alternative explanation for the association of an intact MMR system with cisplatin sensitivity could be that binding of MMR proteins to the drug damage might directly cause activation of the DNA damage response resulting in cell death. This hypothesis is supported by findings of Topping et al. who demonstrated in various colorectal or endometrial cancer cell lines the activation of apoptotic signaling upon treatment with cisplatin. Activation of apoptotic signaling was dependent on MMR proteins and executed via cytochrome c relocalization to the cytoplasm followed by cleavage of caspase-9, caspase-3, and poly(ADP-ribose)polymerase [67]. Furthermore, it was suggested that this MMR protein-dependent response to cisplatin might be independent of the repair function of the MMR proteins [68,69,70]. MMR dependent apoptosis upon the cisplatin treatment might also be executed by direct signaling via p73 and c-Abl [71]. However, regardless of the mechanism of how intact MMR signals apoptosis is, the loss of MMR function can give rise to cisplatin resistance and hence might have an adverse effect on the therapeutic efficacy in cancer therapy.

Futile repair or MMR protein triggered pro-death DNA damage signaling are toxic events as demonstrated in various MMR deficient tumor cell lines derived from colorectal cancer, endometrial carcinoma, embryonic kidney, and ovarian carcinoma, which were found to be more tolerant to cisplatin [57,72,73]. Even though in these cell culture models defective MMR is considered a minor contributor for the cisplatin resistance phenotype [74,75], the preclinical observations still imply that the loss of MMR function might have an adverse effect on cisplatin efficacy in cancer therapy. In patients with ovarian cancer or testicular germ cell tumors, a possible role of MMR deficiency was associated with an acquired resistance towards cisplatin [76,77]. With regards to colorectal cancer, a MMR deficient phenotype is frequently observed, either due to mutations in MMR genes or as a result of hypermethylation of the *MLH1* gene [52,78,79,80]. As an intact MMR system appears to be essential for the linkage of cisplatin damage with initiation of apoptosis, the MMR defect is therefore considered to strongly contribute to the intrinsic cisplatin resistance observed in patients with colorectal cancer.

## 3. The p53-Induced DNA Damage Response and Cellular Resistance to Cisplatin

### 3.1. Activation of Cisplatin-Induced p53-Dependent DNA Damage Signaling

**Figure 4:**
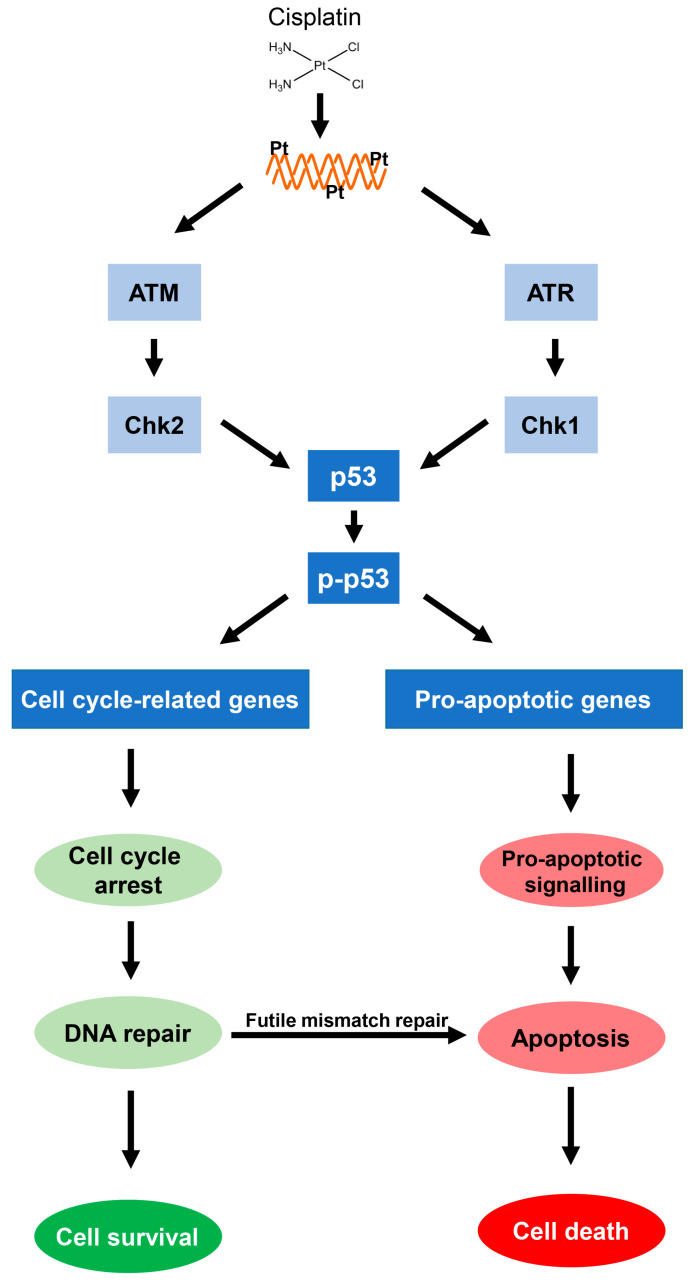
The p53-mediated DNA damage response. Cisplatin-induced DNA damage activates ATM/Chk2 and/or ATR/Chk1, which leads to phosphorylation of p53. Activated p53 induces expression of genes related to the cell cycle control allowing time for DNA repair. If the amount of damage exceeds the cellular repair capacity, apoptosis-related genes will be expressed leading to apoptotic cell death. Futile mismatch repair (MMR) also contributes to cisplatin-induced apoptosis.

In response to cisplatin lesions, cell cycle checkpoints will be activated to delay cell cycle progression, which will facilitate DNA repair. High levels of cisplatin-induced damage, however, might exceed the repair capacity of the cells and in turn trigger apoptosis. It is well established that apoptosis contributes to the cytotoxic action of cisplatin [81]. The exact mechanism of how cisplatin lesions are recognized and the signal becomes transmitted to the DNA damage response is still not entirely clear [82]. It is assumed that the platination damage might cause a block of the replication machinery resulting in replication-mediated DNA double strand breaks (DSBs). Sensing of the DSBs is followed by a series of kinase reactions, which will initiate DNA damage response, promoting either cell survival or cell death (Figure 4) [83,84]. Furthermore, ICLs might be recognized directly by proteins of the Fanconi anemia pathway, as FA proteins could directly sense this type of lesion due to the distortion created in the DNA helix [85]. Following sensing of DNA platination damage, the cells will activate signal transduction via the ATM/Chk2 and/or ATR/Chk1 pathway. As a result, p53 will be phosphorylated and undergoes transient stabilization and activation leading to the transcription of numerous target genes that result in cell cycle arrest or apoptosis [86,87]. ATR seems to be preferentially activated by cisplatin leading to sequential phosphorylation and activation of Chk1 and p53 [13,32,85,88,89]. Chk2 activation following the cisplatin treatment might also occur in an ATM-independent manner [88,90]. Other possible upstream factors of p53 activation upon the cisplatin treatment are the kinases ERK1 and ERK2 [13,91].

As stated, p53 is a key player in the cellular response to cisplatin and controls the down-stream events cell cycle arrest/repair or apoptosis, largely through transcriptional activation of numerous p53 response genes [92]. It has been observed that low levels of cisplatin DNA damage transiently activate p53 favoring cell cycle arrest, while high levels of cisplatin DNA damage lead to sustained p53 activation favoring apoptosis [93]. Cisplatin-induced apoptosis may be triggered through the extrinsic death receptor pathway or the intrinsic mitochondrial pathway. The extrinsic death receptor pathway is mediated through activation of the Fas/FasL system [94], while activation of pro-apoptotic members of the Bcl2 family is involved in the intrinsic mitochondrial pathway [82,90]. In response to chemical agents, p53 has been shown to upregulate the transcription of the pro-apoptotic Bcl2 family genes Bax, Puma, and Noxa [86,95]. In addition to the induction of cell death via p53 signaling, a p53-independent apoptotic response can also be observed following the cisplatin treatment [96,97]. The p53-independent apoptosis might be mediated by the p53-related protein p73, which has been shown to transcriptionally activate p53 target genes and hence induce apoptosis, most likely via activation of the Fas/FasL system [98].

### 3.2. The p53 in Colorectal Cancer Cell Lines and Tissues 

The p53 is mutated in approximately 50% of common human cancers, such as cancers of the breast, colon, and lung [99]. Due to its central role in DNA damage signaling, one would assume that the p53 status determines the response of tumor cells to cisplatin, and mutations in p53 would lead to failure of cell death pathways. An association between the p53 status and response to cisplatin has been investigated in a variety of model systems, but yielded contradictory results. An anticancer drug screen performed by the National Cancer Institute in a panel of 60 cancer cell lines including seven colorectal cancer cell lines demonstrated that the growth inhibition following the cisplatin treatment correlated with the presence of wildtype p53, while the lack of functional p53 was associated with resistance to cisplatin [100]. Similarly, the p53 inactivation rendered astrocytic tumor cells more resistant to the drug [101]. Testis tumor cell lines, which rarely show mutations in the p53 gene, are hypersensitive towards the cisplatin treatment, in part due to intact p53 signaling following the drug treatment [102]. Using the 833K testis tumor cell line, we observed that the siRNA mediated downregulation of p53 resulted in decreased sensitivity towards cisplatin (own unpublished data). However, in a panel of testis testicular cancer cell lines and ovarian cancer cell lines, no correlation between the p53 status and cisplatin response was observed [103,104]. A lack of association between the p53 status and cellular response to cisplatin was also reported for lung adenocarcinoma cell lines [105]. In contrast, in ovarian cancer cells, the loss of functional p53 was associated with increased sensitivity towards cisplatin [106]. Taken together, a clear relationship between the p53 status and cisplatin response has not been observed in cancer cell lines suggesting that the cancer cell type and cellular context have a strong influence on p53-induced DNA damage response. Regarding colorectal cancer cell lines, however, the observations indicate that the lack of functional p53 is related to cisplatin resistance [74,107,108].

In contrast to the findings in pre-clinical studies with cancer cell lines, data obtained in tumor tissues derived from different cancers suggest a clear relationship between the p53 status and response to platinum-based chemotherapy. Several studies have shown that patients with p53 wildtype tumors responded significantly better to cisplatin-based therapy, while inactivation of p53 has been linked to a poor response to the drug [109,110]. Testicular germ cell tumors (TGCT) are particularly sensitive towards the cisplatin treatment, with cure rates above 80%, even when the tumor has metastasized [111]. In contrast to most human cancers, p53 mutations are rare in TGCTs [112,113]. Therefore, it was hypothesized that p53-controlled apoptotic signaling plays an important role in the response of TGCTs to chemotherapeutic drugs, and the lack of p53 mutations explains at least in part the therapeutic response of this type of tumor to cisplatin. Regarding colorectal cancer, one of the most frequently identified gene mutations occurs in the p53 gene, with 40–50% of sporadic colorectal cancer bearing p53 mutations [114,115,116]. For patients with colorectal cancer, it has been observed that tumors with mutant p53 are more chemo-resistant than those with wildtype p53 indicating a correlation between the status of p53 and cancer progression/poorer prognosis.

### 3.3. Impact of Loss of p53 Function in Combination with MMR Deficiency in Colorectal Cancer Cells 

As described, cisplatin resistance in colorectal cancer is mainly caused by alterations of p53-mediated DNA damage response and/or loss of functional MMR. In cell culture models, the defective p53 appears to be the major source of cisplatin resistance, while the MMR defect is considered a minor independent contributor, which induces a 2-fold factor of resistance [74,117]. The most pronounced effect on cisplatin activity in colorectal cancer cells is observed for the loss of p53-mediated DNA damage response combined with a defect in MMR [18,118,119]. 

## 4. Potential Therapeutic Strategies to Circumvent Cisplatin Resistance in Colorectal Cancer Cells

**Figure 5:**
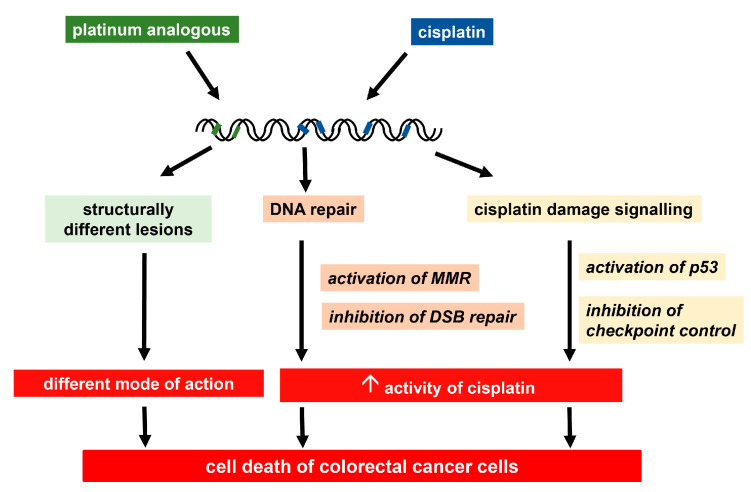
Targeting cisplatin resistance in colorectal cancer cells. Cisplatin-derived Pt (II) and Pt (IV) complexes induce structurally different DNA lesions resulting in a different mode of action compared to cisplatin and hence cell death in cisplatin-resistant colorectal cancer cells. Interfering with the DNA repair via activation of mismatch repair (MMR) or inactivation of double strand break (DSB) repair and targeting cisplatin damage signaling by activation of p53 or inactivation of cell cycle checkpoint control the increase in activity of cisplatin and lead to cell death in colorectal cancer cells.

To expand the therapeutic spectrum of cisplatin to tumor types in which the drug has no significant activity, notably colorectal cancer, several approaches have been proposed, including design of new, improved platinum-based drugs and a combination of cisplatin with modulators of factors underlying the resistance phenotype (Figure 5).

### 4.1. Development of New Platinum-Based Compounds

#### 4.1.1. Oxaliplatin, the First Platinum-Based Compound Approved for Colorectal Cancer Treatment

Many platinum-based compounds have been synthesized and screened for anticancer activity in cisplatin resistant tumor cell lines [11,120,121]. This approach led to the development of carboplatin, which is a direct derivative of cisplatin (Figure 1) [122]. Carboplatin shows less side effects than cisplatin but has no additional activity in resistant tumors and is therefore solely approved for treatment of the same types of cancer. As a third generation platinum drug, the cisplatin analogue oxaliplatin was developed, in which the two ammine ligands have been replaced by a 1,2-diaminocyclohexane (DACH) carrier ligand (Figure 1) [123]. Using various tumor cell lines, it was shown that oxaliplatin has a spectrum of activity which differs from that of cisplatin [124]. Most importantly, pre-clinical investigations revealed that oxaliplatin has a substantial activity in colon cancer cells [125,126].

Oxaliplatin binds to DNA leading to the formation of various structurally different DNA lesions, such as Pt-GG and Pt-AG intrastrand crosslinks and, to a lesser extent, DNA interstrand crosslinks [127,128,129]. The activity of oxaliplatin is primarily a result of induced DNA lesions, but non-DNA targets may also contribute to its toxicity [130]. We and others observed that oxaliplatin induced fewer DNA platination damage than cisplatin, but it induced cell death to a comparable extent indicating that the damage has a higher impact [127,131]. Both cisplatin and oxaliplatin are active by the formation of DNA crosslinks, but oxaliplatin forms lesions on DNA with structures distinct to cisplatin lesions leading to a different gene expression profile and different modes of action [131,132]. In response to oxaliplatin DNA damage, cell death is induced, which is mainly executed by the intrinsic apoptosis pathway, mediated by translocation of BAX to the mitochondria followed by cytochrome C release into the cytoplasm and activation of caspase 3 [133].

In strong contrast to cisplatin damage, oxaliplatin-induced DNA lesions are not recognized by MMR proteins [134] and oxaliplatin, unlike cisplatin, showed a substantial activity in MMR defective cells [135,136], which makes the cytotoxicity of oxaliplatin independent of the cellular MMR function. This explains the pre-clinical observation that oxaliplatin has a substantial activity in colon cancer cells lines, which are often deficient in MMR and hence resistant to cisplatin. The findings obtained in cell culture models suggested that oxaliplatin might possess clinical activity against intrinsically cisplatin resistant colorectal cancer, which is frequently characterized by a MMR deficient phenotype due to defects in MMR genes [137,138]. Various clinical studies confirmed that oxaliplatin showed activity in colorectal cancer [11,139,140,141] leading to a world-wide approval of this drug for the treatment of metastatic colorectal cancer [120,142,143].

#### 4.1.2. Design of Further Platinum Analogues with Possible Activity in Colorectal Cancer

In addition to the three platinum-based drugs cisplatin, carboplatin, and oxaliplatin which achieved world-wide clinical approval, numerous more platinum compounds have been designed and are still being synthesized to expand the therapeutic spectrum with respect to colorectal cancer and other cancers, in which cisplatin has no significant clinical activity (Table 1) [120,121,122].

The Pt (II) complex picoplatin was designed as a drug that sterically hinders the binding of thiol-containing molecules such as glutathione and metallothionein, which sequester cisplatin in the cytoplasm before it can reach its target DNA in the nucleus [144,145]. Therefore, picoplatin is less susceptible to detoxification by intracellular thiols, and it was shown that picoplatin retained activity against a wide range of cisplatin resistant tumor cell lines including colorectal cancer cells [146,147] as well as antitumor activity in vivo in tumor xenografts [148]. Picoplatin has undergone clinical testing in a variety of cancers, including prostate, lung or colorectal cancer [11,120,149]. Even though it has shown some evidence of antitumor activity in phase 2 trials, it failed to demonstrate a survival advantage in a phase 3 trial and gradually all ongoing trials were discontinued.

Satraplatin belongs to a series of platinum (IV) complexes with lipophilic ligands, which have been developed to overcome the poor bioavailability of cisplatin, carboplatin, and oxaliplatin [145]. Satraplatin acts as a prodrug that, upon reduction in the cells, releases a Pt (II) complex, which induces intra- and interstrand crosslinks in the DNA [150]. Due to its high stability and bioavailability it can be administered orally. Unlike cisplatin, satraplatin showed in vitro cytotoxicity in colorectal cancer cell lines and possessed some in vivo antitumor activity in human xenografts of colon carcinoma cells and mouse tumor models [57,151,152]. Based on the pre-clinical observations, satraplatin entered clinical trials and showed antineoplastic activity. However, satraplatin failed to show a clinically relevant benefit with regards to the overall survival or progression-free survival and therefore has not gained clinical approval [149,153,154,155].

BBR3464, a trinuclear platinum (II) complex, forms flexible, “long distance“ ICLs over a range up to six DNA base pairs, and these unique DNA lesions are thought to mediate its activity [156,157]. In vitro studies revealed a pattern of BBR3463 cytotoxicity, which was markedly different from that of cisplatin, and it was active in cisplatin resistant colorectal cancer cells [158,159]. BBR3464 entered clinical trials for a variety of tumor types but showed a relatively poor response rate and hence failed in early clinical development [160,161].

In addition to these platinum complexes, numerous more cisplatin derivatives have been developed. Iproplatin, an octahedral Pt (IV) drug, has entered clinical trials for a variety of solid human cancers [120]. However, as iproplatin was either largely inactive in the majority of cancers, including colorectal cancer or did not show any benefit compared to cisplatin, it was discontinued in clinical evaluations [162,163,164]. The Pt (II) complex phenanthriplatin induces monofunctional adducts at guanine bases, which interfere with the normal function of RNA polymerase II that ultimately will lead to apoptosis in cancer cells [165]. Phenanthriplatin has been shown to be more cytotoxic than cisplatin and oxaliplatin in a number of cell lines derived from cancers of the lung, cervix, bones, and prostate. Moreover, it showed a cytotoxic efficiency in the colorectal cancer cell line HT29 comparable to that of oxaliplatin [166]. However, to date, no evaluation of phenanthriplatin in clinical trials has been reported. Aroplatin, a structural analogue of oxaliplatin with liposomal encapsulation, which results in enhanced uptake and hence increased toxicity in colorectal cancer cells compared to cisplatin [167], was evaluated in a phase 2 trial with patients with therapy-refractory colorectal cancer [168]. Even though the response was promising and antitumor activity was observed, no clinical approval has yet been achieved. A cytostatic effect in colorectal cancer cell lines was also observed for the Pt (II) complex kiteplatin [169] and for a variety of different Pt (IV) derivatives containing a cyclohexane-1R,2R-diamine carrier ligand or two axial PhB ligands [170,171], but in addition to pre-clinical observations no clinical data are available. Three asymmetric platinum (IV) bis-carboxylate prodrugs showed cytotoxicity in the colorectal cancer cell lines HT29 and HCT116 and antiproliferative activity in a colorectal cancer animal model [152], but no information is available as to the activity in colorectal cancer tissue.

Altogether, even though some of the newer platinum analogues showed in vitro toxicity in cancer cells, among them colorectal cancer cell lines, and in vivo activity in animal models with tumor xenografts, they either did not reach clinical evaluation or failed in early clinical development and were discontinued. Hence, in addition to the approved drugs cisplatin, carboplatin, and oxaliplatin, no additional platinum drug has yet received worldwide approval for cancer treatment, and oxaliplatin is still the only platinum complex approved as a chemotherapeutic drug for colorectal cancer. 

### 4.2. Strategies to Target Cisplatin Resistance Factors in Colorectal Cancer Cells

Combinations of cisplatin with compounds that interfere with specific cisplatin resistance factors have been tested in various preclinical models of cancer. Notably, p53 and p53-mediated DNA damage response might be used as targets for biochemical modulators in colorectal cancer cells.

#### 4.2.1. Restoration of Functional p53 Response in Colorectal Cancer Cells

As the lack of a functional p53 response in tumor cells is associated with resistance to chemotherapeutic drugs, re-activation of the p53 pathway is another therapeutic strategy against cancer [172]. About 50% of cancers harbor mutations in the TP53 gene, while in tumors retaining wildtype p53, MDM2 plays an important role in regulating the p53 protein. MDM2 is a major physiological antagonist of p53 promoting its proteasomal degradation. In cancer, MDM2 is often highly expressed and hence suppresses the activity of p53 [173]. Therefore, the interaction of p53 with MDM2 might be used as a target for anti-cancer therapies [174,175]. The bicyclic lactam SYNAP is one of these novel p53 activating agents, which showed a synergistic effect on cisplatin-induced growth inhibition in the HCT116 colorectal cancer cell line due to disruption of the interaction of p53 with MDM2 [176]. To date, no in vivo data are reported for SYNAP, but the observations may serve as the foundation for the design of further, more effective anticancer drugs targeting the p53-MDM2 interaction.

#### 4.2.2. Exploiting DNA Repair Pathways to Improve Cisplatin Activity

Effects of chemotherapeutic drugs are also influenced by the efficiency of DNA repair. Exploiting DNA repair might be another strategy in cancer therapy. Kopa et al. observed an increase in cisplatin cytotoxicity by inhibiting the repair of DSBs, which are assumed to arise during the processing of cisplatin-induced ICLs [177]. DSBs are repaired mainly by the NHEJ pathway, which seems to be intact in colorectal cancer contributing to intrinsic resistance. The NHEJ inhibitors SCR7 and NU7441 showed a sensitizing effect to cisplatin in the colon cancer cell line LoVo, but the sensitizing effect was rather small and to date has been observed solely in vitro [177].

The loss of the MMR pathway due to silencing of the MLH1 gene as a result of promoter methylation is frequently observed in colorectal cancer and associated with resistance towards cisplatin. Therefore, a combination of de-methylating agent with cisplatin might have the potential to reverse the resistance phenotype and hence increase the cytotoxic efficiency of the drug in colorectal cancer cells. Indeed, the de-methylating agent decitabine sensitized human colon tumor xenografts to cisplatin resulting in increased chemotherapeutic efficacy [178]. The de-methylating agents azacitidine and decitabine are also being tested in combination with platinum drugs in clinical trials in various tumor types including colorectal cancer [179,180].

#### 4.2.3. Disruption of Signal Transduction 

Another strategy to overcome tumor cell resistance is the combination of cisplatin with a putative modulator of signal transduction. The cisplatin treatment results in cell cycle arrest, mediated by the serine/threonine kinases Chk1 and Chk2 [181], which phosphorylate p53. Inhibition of Chk1/2 may disable cell cycle checkpoints and therefore allow the cells to progress through the cell cycle despite the presence of DNA damage, which ultimately results in apoptosis [182].

To interfere with Chk1/Chk2-mediated DNA, the damage response might therefore increase the efficacy of cisplatin in cancer cells. The impact of different Chk1/Chk2 kinase inhibitors on cisplatin toxicity has been investigated in various cancer cells lines, however, with mixed results [183,184]. In colorectal cancer cell lines, though, inhibition of Chk1/2 potentiated the activity of cisplatin, which was due to alterations of cell cycle progression [185,186,187]. Clinical investigations are currently under way to evaluate whether Chk1/Chk2 kinase inhibitors will sensitize different tumor types to the cytotoxic effects of cisplatin and other chemotherapeutic drugs [188,189,190].

An alternative option to improve the activity of cisplatin in cancer cells was reported for the combination of cisplatin with the antidepressant desipramine as a pharmacological adjuvant [159]. Desipramine was found to increase the cytotoxicity of cisplatin in the colorectal cancer cell line HCT116, this sensitization was most likely due to interfering with p53-mediated signal transduction and apoptosis [159].

## 5. Conclusions

Cisplatin is a commonly utilized potent chemotherapeutic agent used for the treatment of a variety of cancers, including testicular, ovarian, head and neck, bladder, and lung cancer. However, its clinical success is limited by tumor cell resistance, which might be acquired during cycles of therapy or intrinsic to the cancer. With regards to colorectal cancer, cisplatin resistance is mainly caused by disruption of p53-mediated DNA damage response and/or loss of MMR function. Different strategies can be envisaged to overcome the resistance phenotype. A variety of new platinum analogues with a different mode of action compared to cisplatin have been tested in various cancer cell cultures and animal models. Various platinum-based compounds showed promising activity in a clinical setting, but so far only oxaliplatin has gained world-wide approval for the treatment of metastatic colorectal cancer. A combined application of cisplatin with biochemical modulators of p53-mediated DNA damage response or MMR function frequently led to a favorable response in colon cancer cell lines and pre-clinical tumor models of colorectal cancer. To date, no successful clinical trials are reported, but the information gained in a pre-clinical setting can serve as a basis for future research to identify more effective treatment regimens, especially for colorectal cancer.

## Figures and Tables

**Table 1:** Platinum-based compounds and examples of clinical trials with platinum-based compounds. NCT#: Clinical trial ID.

Platinum-Based Compounds	Clinical Trials of Platinum-Based Compounds
Carboplatin	World-wide approval for treatment of various solid tumors, including ovarian, lung, head/neck, bladder, and cervical cancers
Oxaliplatin	World-wide approval for treatment of **metastatic colorectal cancer**
Picoplatin	NCT00465725: Phase 1 trial for various solid tumors, including **colorectal cancer** NCT00478946: Phase 1/Phase 2 trial for **colorectal cancer**
Satraplatin	NCT00473720: Phase 1 trial for various advanced cancers
BBR3464	NCT00014547: Phase 2 trial for lung cancer NCT00024362: Phase 2 trial for pancreatic cancer
Aroplatin	NCT00316511: Phase 1 trial for advanced solid malignancies or B-Cell lymphoma NCT00081549: Phase 1/Phase 2 trial for pancreatic cancer NCT00081536: Phase 1/Phase 2 trial for **advanced colorectal cancer** NCT00043199: Phase 2 trial for **metastatic colorectal cancer** NCT00057395: Phase 1/Phase 2 trial for advanced solid malignancies including **colorectal neoplasms** NCT00004033: Phase 2 trial for malignant pleural mesothelioma
Iproplatin	No information on clinical cancer trials
Kiteplatin	No information on clinical cancer trials
Phenanthriplatin	No information on clinical cancer trials

Bold: highlight colorectal cancer.

## Abbreviations

ICL	interstrand crosslink
MMR	mismatch repair
NER	nucleotide excision repair
SNP	single nucleotide polymorphism
TGCT	testicular germ cell tumors
DSB	double strand break
